# High-Gain Metasurface in Polyimide On-Chip Antenna Based on CRLH-TL for Sub-Terahertz Integrated Circuits

**DOI:** 10.1038/s41598-020-61099-8

**Published:** 2020-03-09

**Authors:** Mohammad Alibakhshikenari, Bal S. Virdee, Chan H. See, Raed A. Abd-Alhameed, Francisco Falcone, Ernesto Limiti

**Affiliations:** 1 Electronic Engineering Department, University of Rome “Tor Vergata”, Via del Politecnico 1, 00133 Rome, Italy; 2 London Metropolitan University, Center for Communications Technology & Mathematics, School of Computing & Digital Media, London, N7 8DB UK; 3 School of Engineering & the Built Environment, Edinburgh Napier University, 10 Colinton Road, Edinburgh, EH10 5DT UK; 4 School of Engineering, University of Bolton, Deane Road, Bolton, BL3 5AB UK; 5 School of Electrical Engineering & Computer Science, University of Bradford, Bradford, BD7 1DP UK; 6 Electrical and Electronic Engineering Department, Public University of Navarre, 31006 Pamplona, Spain

**Keywords:** Electrical and electronic engineering, Electronic devices

## Abstract

This paper presents a novel on-chip antenna using standard CMOS-technology based on metasurface implemented on two-layers polyimide substrates with a thickness of 500 *μ*m. The aluminium ground-plane with thickness of 3 *μ*m is sandwiched between the two-layers. Concentric dielectric-rings are etched in the ground-plane under the radiation patches implemented on the top-layer. The radiation patches comprise concentric metal-rings that are arranged in a 3 × 3 matrix. The antennas are excited by coupling electromagnetic energy through the gaps of the concentric dielectric-rings in the ground-plane using a microstrip feedline created on the bottom polyimide-layer. The open-ended feedline is split in three-branches that are aligned under the radiation elements to couple the maximum energy. In this structure, the concentric metal-rings essentially act as series left-handed capacitances *C*_*L*_ that extend the effective aperture area of the antenna without affecting its dimensions, and the concentric dielectric rings etched in the ground-plane act as shunt left-handed inductors *L*_*L*_, which suppress the surface-waves and reduce the substrates losses that leads to improved bandwidth and radiation properties. The overall structure behaves like a metasurface that is shown to exhibit a very large bandwidth of 0.350–0.385 THz with an average radiation gain and efficiency of 8.15dBi and 65.71%, respectively. It has dimensions of 6 × 6 × 1 mm^3^ that makes it suitable for on-chip implementation.

## Introduction

Antenna is the key component to enable wireless communication however their physical size is a function of the operating frequency. Applications of on-chip antennas is therefore limited to high-frequencies due to the large size of antenna at lower frequencies. Off chip antennas however offer the benefit of radiation efficiency as they can be implemented on low-loss dielectric substrates^1–6^..

Currently, antennas for front-end transceivers can be realised using three different methods, which include: (i) Antenna-in-Package (AiP), where the antenna is embedded in the IC’s packaging; (ii) Antenna-on-Chip (AoC), where the antenna is realized on substrate; and (iii) this is a hybrid of AoC and AiP, where the radiating element is realized off-chip. Wire bonding and flip chip are the two commonly used interconnections techniques employed in AiP to connect the die and the antenna^7^. These types of interconnects are highly lossy at high frequencies due to impedance mismatch. The only viable solution to overcome this loss is by using on-chip antenna, which should significantly reduce the manufacturing cost of system-on-chip (SoC).

The development of a truly efficient cost effective on-chip antenna is a challenging endeavour. The main challenges are attributed to (1) low resistivity of dielectric substrates, which is approximately 10 ohm-cm, contributes to substrate loss of 85% whereas metallization loss is only 15%; (2) high permittivity substrate confines most of the electromagnetic energy in the substrate rather than being radiated into free-space, which adversely affect radiation efficiency^8^; (3) no specific design rule for antenna design in standard technologies for on-chip antennas. Also, the typical metallization thickness using standard technologies results in poor radiation because thin metal layer has a high resistance; and (4) on-chip antenna’s radiation characteristics cannot be measured in an anechoic chamber unless the chip is mounted on a special test fixture. Mounting the chip on a test fixture can results in undesirable radiation because of interference from nearby circuit components.

In the paper it is shown that the issues mentioned above afflicting on-chip antennas can be reduced by applying the 2D composite right/left-handed (CRLH) metamaterial transmission line (TL) known as metasurface concept in the development of the antenna. The performance of the proposed technique when compared with the conventional on-chip antenna designs shows improvement in the impedance bandwidth, radiation gain and efficiency. In addition, the proposed technique has no effect in the dimensions of the antenna. These results show the promise of metasurface on-chip antenna for application in sub-THz integrated circuits.

## Overcome the Challenges and Increase the On-Chip Antenna Radiation Properties

**Figure 1:**
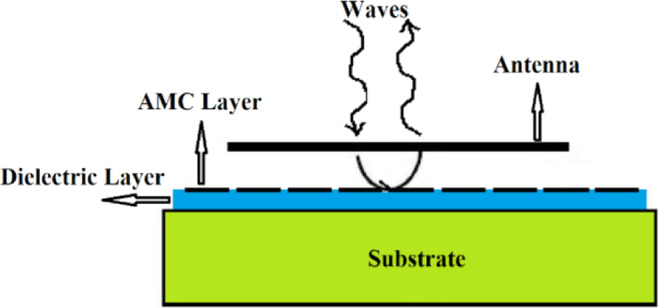
Wave incident on artificial magnetic conductor surface.

Currently on-chip antennas possess a poor radiation efficiency and gain characteristics. One technique to enhance radiation efficiency is achieved by inserting an artificial magnetic conductor (AMC) between the on-chip antenna and the lossy substrate. The radiation element is located above the AMC layer, as shown in Fig. 1 ^7,9^. This technique can effectively eliminate back-lobe radiation.

Reflection-coefficient of a plane TEM wave incident on Perfect Electric Conductor (PEC) is −1, which demonstrate that reflected wave will cancel the incident wave. On the other hand, the AMC plane indicates a reflection coefficient of +1, which means that the phase of the reflected wave is in phase of the incident wave. Therefore, with an AMC plane we can produce constructive phase reflections with the incident wave at across a finite operating frequency band. The consequence of this is enhanced radiation properties.

Numerous AMC structures have been explored to date in order to improve the radiation efficiency and bandwidth of on-chip antennas^10–13^, which includes proton implantation and micromachining^14,15^. These AMC structures require complex manufacturing steps and hence are highly expensive for mass production.

**Figure 2:**
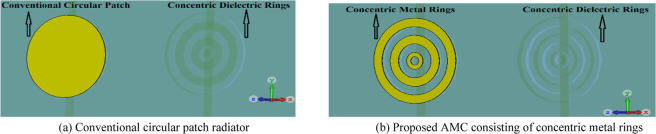
Geometry of the proposed AMC structure and the conventional circular patch. (**a**) Conventional circular patch radiator. (**b**) Proposed AMC consisting of concentric metal rings.

**Figure 3:**
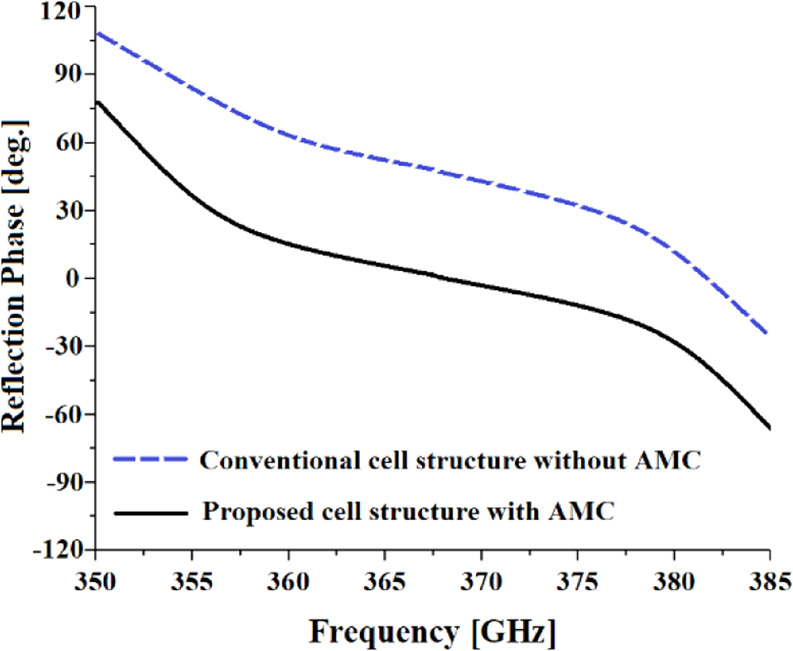
Reflection phase of the wave incident on AMC structure.

**Figure 4:**
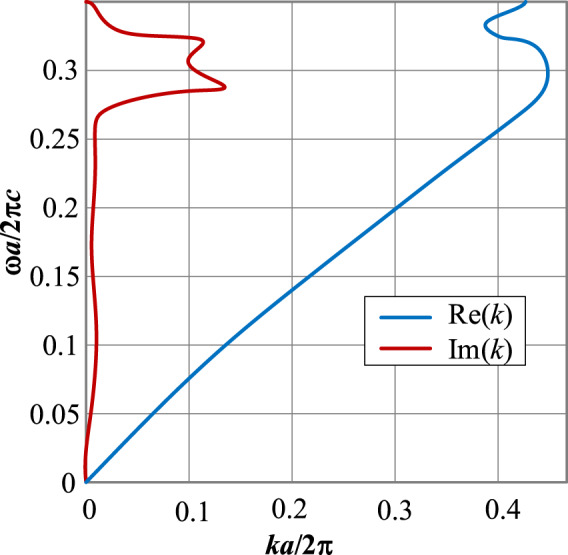
Real and imaginary parts of the dispersion diagram where *k* is the wave number and *a* is the gap between the radiation elements.

In this paper an artificial magnetic conductor (AMC) is designed using metasurface structure as a 2D composite right/left-handed (CRLH) metamaterial transmission line (TL), which was realized by etching concentric dielectric rings in the ground-plane of a polyimide substrate that are located under the radiating element comprising concentric metal rings constructed on the top substrate layer, as shown in Fig. 2. To accurately characterize the reflection phase of the incident wave on the metasurface structure a 3D full-wave EM solver based on finite element method (CST Microwave Studio) was used. The simulated reflection phase of the wave incident on the proposed AMC structure is compared with a conventional circular patch in Fig. 3. The results clearly show that with CRLH metamaterial-based AMC structure the reflection phase significantly drops and is around zero between 0.360 THz to 0.375 THz, which is a region where the losses are minimum and therefore optimum radiation achieved. The dispersion diagram of the proposed metamaterial structure in Fig. 2(b) is depicted in Fig. 4. It shows the negative group velocity is centered at around *ωa*/2π*c* = 0.3, which corresponds to approximately 365 GHz. The diagram reveals the range of the negative group velocity is approximately between 0.28 to 0.32.

**Figure 5:**
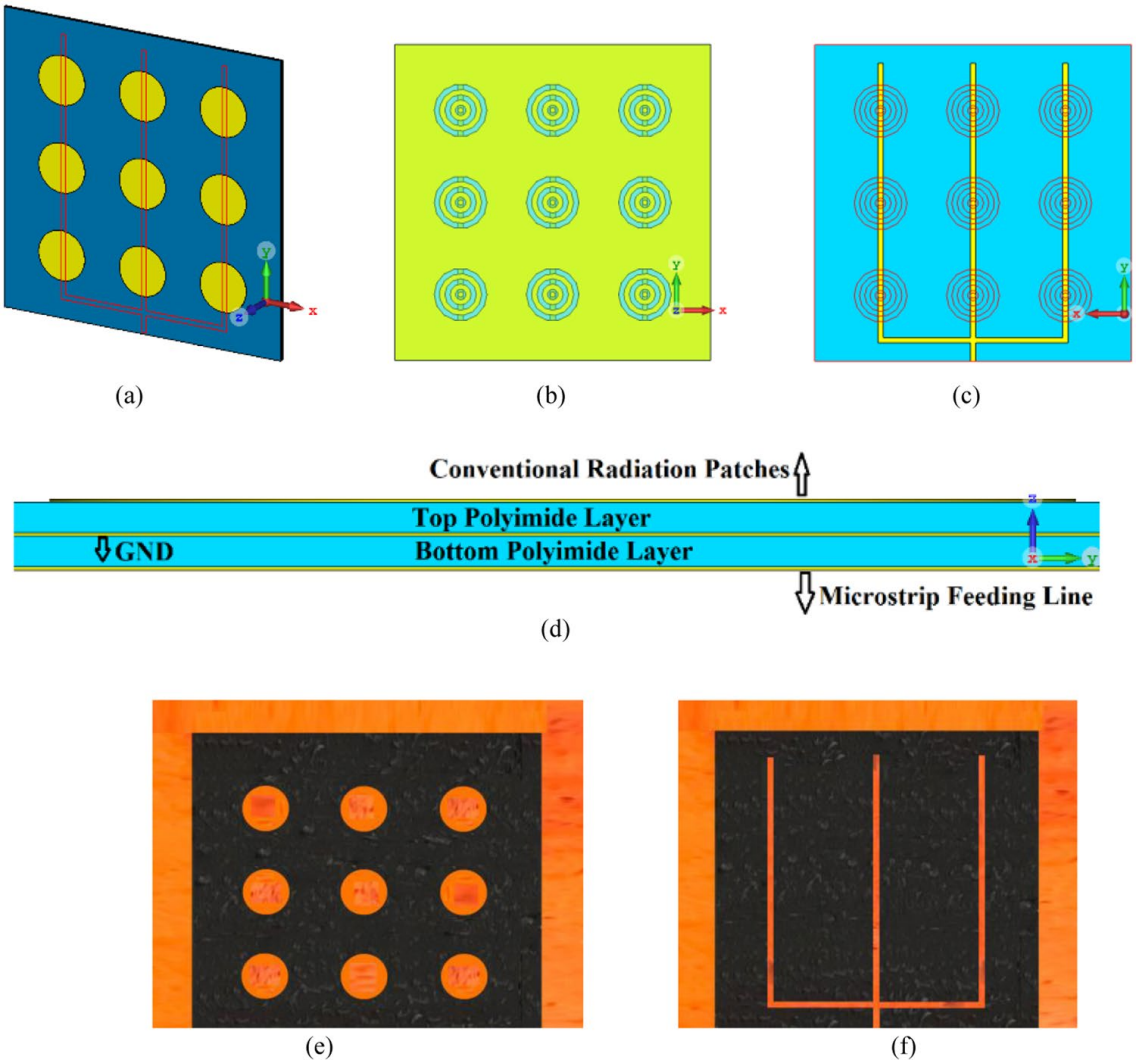
(**a**) Conventional circular radiation patches (yellow) on the top of the substrate and the CPW feedlines (red lines) on the underside of the bottom layer, (**b**) Concentric dielectric rings etched in the middle layer and located under the circular patches along with CPW open-circuited feedline located under the concentric dielectric rings, (**c**) back view of bottom layer showing CPW feedlines, and (**d**) cross-section of the antenna structure comprising two metallized polyimide layers, (**e**) Fabricated prototype, top view, and (**f**) Fabricated prototype, back view.

**Figure 6:**
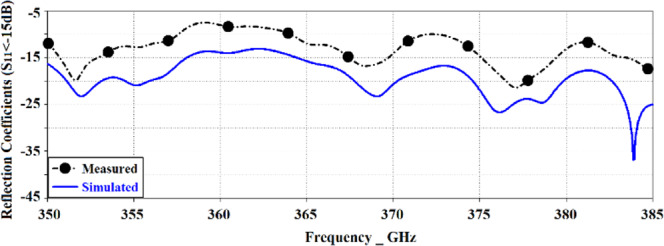
Simulated and measured reflection-coefficient responses of the proposed antenna array with circular patches.

**Figure 7:**
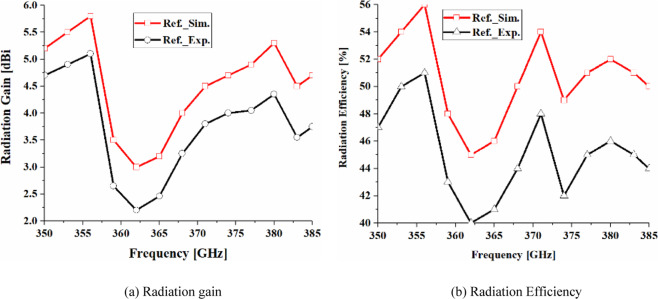
Simulated and measured radiation gain and efficiency of the antenna array with conventional circular patches as a function of frequency.

The proposed antenna was used to construct an antenna array comprising 3 × 3 circular radiating patches, as shown in Fig. 5. The array was constructed on two layers of polyimide stacked on top of each other where radiating elements are constructed on the top layer, the AMC structure constructed in the middle ground-plane layer, and the feeding network implanted in the underside of the bottom polyimide layer. Thickness of the polyimide substrate is 500 *μ*m with a metallization layer of 3 *μ*m thickness. The feedline is split in three open-ended branches that are located under the radiation elements. The antennas are excited by coupling electromagnetic energy through the gaps of the concentric dielectric rings using a microstrip feedline. The proximity between the feedline in the bottom layer and ground-plane causes image current to flow in the ground-plane that causes loss of energy in the form of heat. So, preferring CPW over microstrip line feeding is an excellent choice. Concentric dielectric rings in the ground-plane reduces substrate loss and suppresses surface waves which leads to enhanced bandwidth and radiation properties. The dimensions of the 3 × 3 antenna array shown in Fig. 5 is 6 × 6 × 1 mm^3^. The characteristics of the antenna was measured using a compact antenna test range as described in^16^. The simulated and measured reflection-coefficient responses of this antenna in Fig. 6 shows the antenna exhibits a bandwidth from 0.350 THz to 0.385 THz for S_11_ <−10 dB with a notch-band from 0.3575 THz to 0.3640 THz. Figure 7 shows the array’s measured radiation gain and efficiency vary from 2.2 dBi to 5.1 dBi and 40.34% to 51.67%, respectively, over its operating frequency band. The discrepancy between the measured and simulated results is attributed to the unknown dielectric loss-tangent of the polyimide layer over the frequency band of interest in the foundry’s design kit as well as unaccounted manufacturing tolerances.

**Figure 8:**
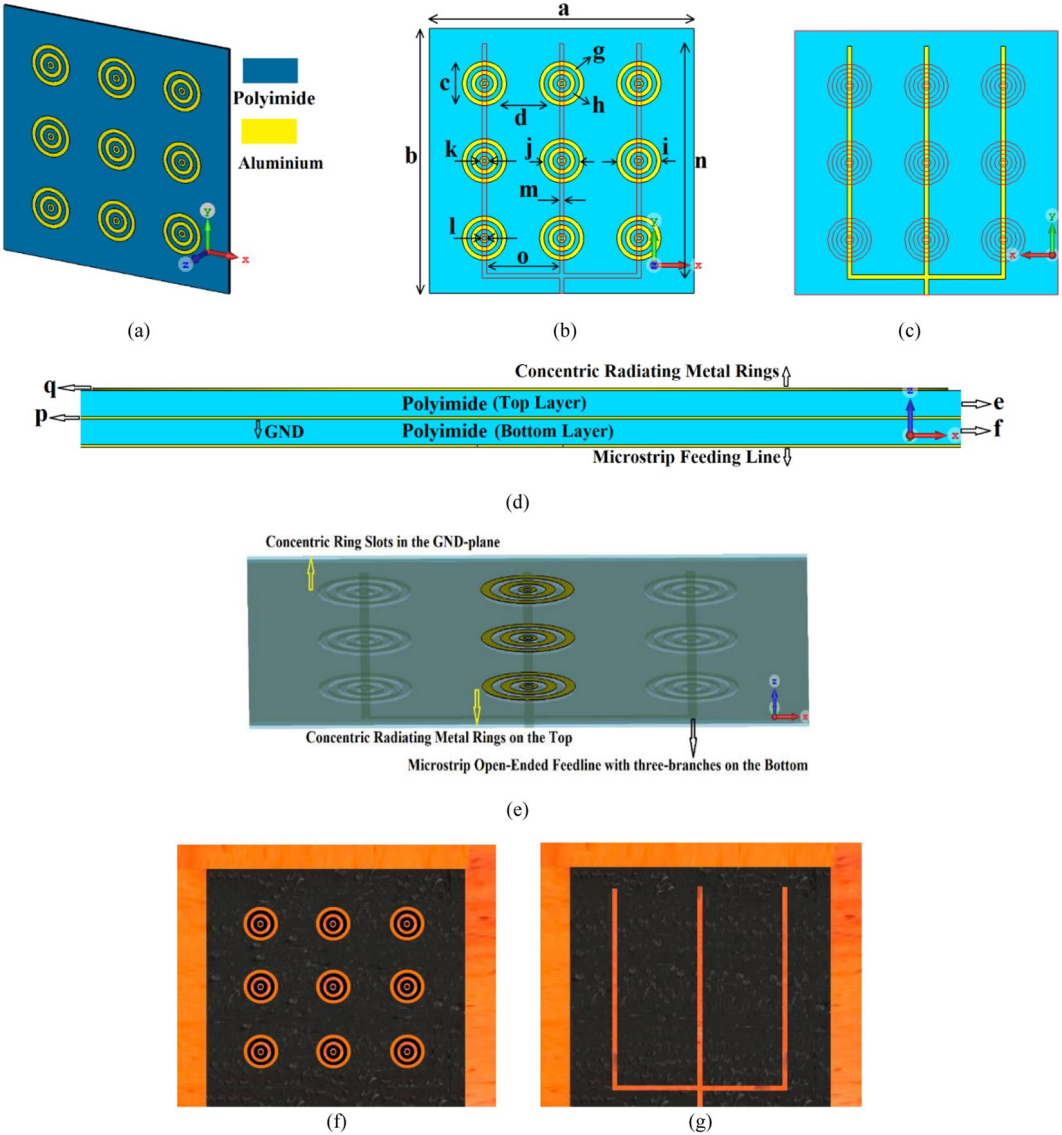
(**a**) Isometric view of the on-chip antenna with AMC metasurface structure consisting of concentric radiation rings on the top of a standard polyimide substrate, (**b**) Concentric dielectric rings antennas (shown in yellow) in the middle layer and located under the concentric radiation rings along with CPW open-circuited feedline located under the concentric dielectric rings, (**c**) Back view showing the CPW feedline in ‘yellow’. The concentric dielectric rings (shown in red) are fabricated in the middle layer, (**d**) Cross-section view of the metasurface antenna containing two metallized polyimide layers, (**e**) Top view showing the concentric radiating metal rings in ‘yellow’, concentric dielectric ring slots in the middle layer, and CPW open-circuited three branched feed line on the underside of the bottom layer, (**f**) Fabricated prototype, top view, and (**g**) Fabricated prototype, back view.

The circular radiation patches were replaced with concentric rings, as shown in Fig. 8. The dimensions of this structure are tabulated in Table 1. In this structure the concentric radiation rings essentially act as series left-handed capacitances (C_L_) that extend the effective aperture area of the antenna without affecting its dimensions and the concentric dielectric rings etched in the middle layer ground-plane act as shunt left-handed inductances (L_L_), which suppress the surface-waves and reduce the substrates losses that lead to improved bandwidth and radiation properties. The structure possesses the right-handed parasitic effects that can be seen as shunt right-handed capacitance (C_R_) and series right-handed inductance (L_R_). The shunt right-handed capacitance C_R_ are mostly come from the gap capacitance between the patch and the ground plane, and the series right-handed inductance L_R_ is created by unavoidable currents that flowing on the patches, which indicates that these capacitance and inductance cannot be ignored. Therefore, the overall structure behaves like a 2D composite right/left-handed (CRLH) metamaterial transmission line (TL) known as metasurface.

**Table 1:** Structural Parameters Of The On-Chip Antenna Array.

Antenna’s size (a × b)	6 × 6 mm^2^
Circular patch (c)	0.5 mm
Patch spacing (d)	0.75 mm
Thickness of the polyimide layers (e & f)	5 mm
Width of concentric metal rings (g)	0.1 mm
Width of concentric ring slots (h)	0.1 mm
Max. radius of concentric metal rings (i)	0.5 mm
Max. radius of concentric ring slots (j)	0.4 mm
Min. radius of concentric metal rings (k)	0.1 mm
Min. radius of concentric ring slots (l)	0.1 mm
Number of concentric metal rings	3
Width of feedline (m)	0.1 mm
Length of feedline branches (n)	5.3 mm
Space between the feedline branches (o)	1.5 mm
Thickness of metallic conductor (p & q)	0.003 mm

**Figure 9:**
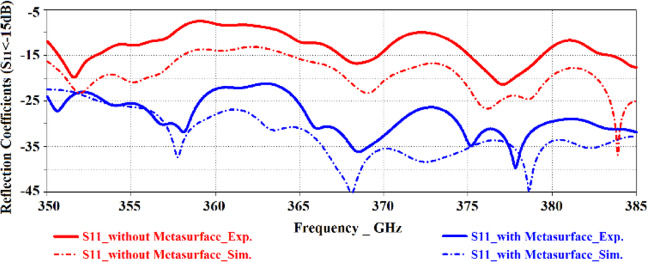
Simulated and measured reflection-coefficient responses of the proposed on-chip antenna ‘with’ and ‘without’ the metasurface.

**Figure 10:**
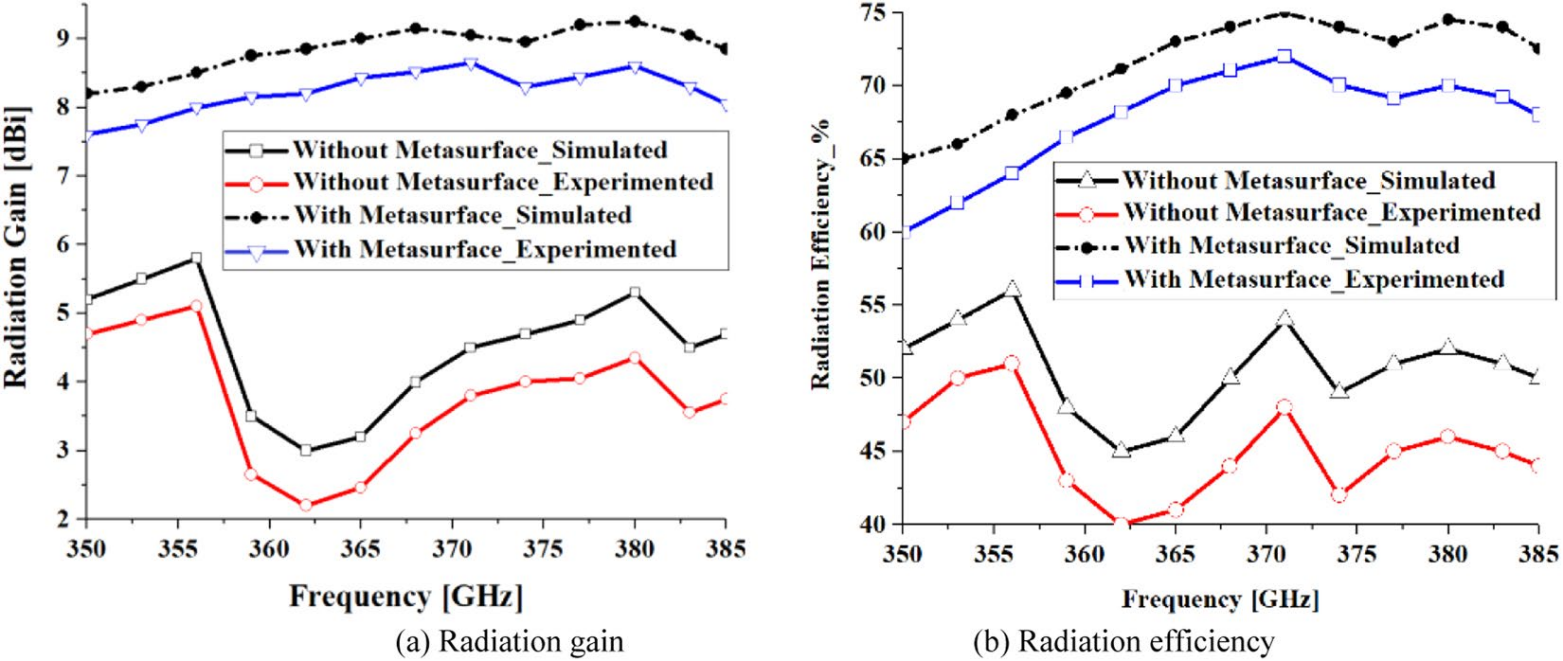
Simulated and measured radiation gain and efficiency curve plots of the proposed on-chip antennas ‘with’ and ‘without’ metasurface.

Figure 9 shows the antenna with metasurface structure exhibits a bandwidth from 0.350 THz to 0.385 THz for S_11_ <−20 dB. The average impedance match of this antenna with the metasurface is −30 dB that is almost two-fold than without the metasurface, which has an average impedance match of −12.5 dB. Figure 10. shows that the radiation gain and efficiency of the antenna change from 7.58 dBi to 8.65 dBi, and 60.85% to 72.47%, respectively. The average gain and efficiency are 8.15 dBi and 65.71%, respectively, which constitutes an improvement of 5 dBi and 20.42% when compared with no metasurface. The radiation properties are listed in Table 2. The results shows that, after apply the metasurface concept the effective aperture area of the on-chip antenna has extended and also the surface-waves and the substrates losses have suppressed without enhancing its dimensions^17^, which have caused to improve the on-chip antenna performance parameters such as impedance match, impedance bandwidth, radiation gain, and radiation efficiency.

**Table 2:** Measured radiation characteristics of the proposed on-chip antenna.

	Without Metasurface	With Metasurface
Min. gain	2.2 dBi	7.58 dBi
Max. gain	5.1 dBi	8.65 dBi
Average gain	3.15 dBi	8.15 dBi
Average improvement	**5.0 dBi**	
Min. efficiency	40.34%	60.85%
Max. efficiency	51.65%	72.47%
Average efficiency	45.29%	65.71%
Ave. improvement	**20.42%**	

In Table 3, the performance of the proposed on-chip antenna is compared with other types mm-waves and terahertz antennas recently publications. The comparison shows the proposed antenna operates at a much higher frequency and has comparable gain to^18^ but its efficiency is less. Its dimensions are larger than other antennas. However, the proposed antenna is less complex to implement. It is viable candidate for applications in terahertz integrated circuits.

**Table 3:** Specifications of the proposed on-chip antenna compared with the literature.

Refs.	Antenna Type	Frequency Band (GHz)	Gain (dBi)	Efficiency (%)	Dimensions (*λ*_0_ @ 350 GHz)	Process
^10^	Bowtie-slot	90–105	Max. −1.78	—	0.82 × 0.36 × 0.75	IHP 0.13-μmBi −CMOS
^11^	Differential-fed circularly polarized	50–70	Max. −3.2	—	1.75 × 1.75 × 0.35	0.18-μm
^12^	Ring-shaped monopole	50–70	Max. 0.02	Max. 35	—	CMOS 0.18-μm
^13^	Circular open-loop	57–67	Max. −4.4	—	2.10 × 2.10 × 0.35	CMOS 0.18-μm
^18^	Loop antenna	65–69	Max. 8	Max. 96.7	0.81 × 1.45	CMOS 0.18-μm
^19^	AMC embedded squared slot antenna	15–66	Max. 2	—	1.68 × 1.28	CMOS 0.09-μm
^20^	Monopole	45–70	Max. 4.96	—	2.27 × 2.25 × 0.29	Silicon CMOS
^21^	Dipole antenna	95–102	Max. 4.8	—	—	Bi-CMOS
^22^	Tab monopole	45–75	Max. 0.1	Max. 42	1.75 × 1.16	Standard CMOS Silicon
^23^	Transmitter and receiver modules	218–246	Average 8.5	—	3.19 × 0.68	130-nm SiGe HBT Technology
^24^	Metamaterials and dielectric resonators	>450	Max. 4.5	Max. 45.7	0.46 × 0.46 × 0.15	Standard CMOS
^25^	Monopole antenna	~300	Max. 1.72	—	0.35 × 0.35 × 0.09	InP 50- μm Substrates
**This paper**	Metasurface & EM coupled feed mechanism	>350	Min. 7.58	Min. 60.85	7.0 × 7.0 × 1.16	Standard 500-μm Polyimide

## Conclusion

A novel technique based on 2D CRLH metamaterial transmission line known as metasurface is described to design an antenna for on-chip applications operating at sub-terahertz frequency. The antenna was implemented on two layers of polyimide substrates using artificial magnetic conductor (AMC) structure. The antenna was excited using an open-circuited feedline located on the bottom polyimide layer where the electromagnetic signal is coupled to the antenna through the AMC structure. The AMC comprises concentric dielectrics rings constructed in the ground-plane sandwiched between the two-polyimide substrates, which has caused to suppress the surface waves and reduce substrates losses. The radiation elements located on the top polyimide layer are composed of concentric metal rings, which are realized based on the metasurface concept to extend the antenna effective aperture area. The antenna is relatively easy to manufacture and cost effective for mass production.
