# Author Correction: High-Gain Metasurface in Polyimide On-Chip Antenna Based on CRLH-TL for Sub-Terahertz Integrated Circuits

**DOI:** 10.1038/s41598-020-69882-3

**Published:** 2020-08-07

**Authors:** Mohammad Alibakhshikenari, Bal S. Virdee, Chan H. See, Raed A. Abd-Alhameed, Francisco Falcone, Ernesto Limiti

**Affiliations:** 1 Electronic Engineering Department, University of Rome “Tor Vergata”, Via del Politecnico 1, 00133 Rome, Italy; 2 London Metropolitan University, Center for Communications Technology & Mathematics, School of Computing & Digital Media, London, N7 8DB UK; 3 School of Engineering & the Built Environment, Edinburgh Napier University, 10 Colinton Road, Edinburgh, EH10 5DT UK; 4 School of Engineering, University of Bolton, Deane Road, Bolton, BL3 5AB UK; 5 School of Electrical Engineering & Computer Science, University of Bradford, Bradford, BD7 1DP UK; 6 Electrical and Electronic Engineering Department, Public University of Navarre, 31006 Pamplona, Spain

Correction to: *Scientific Reports*10.1038/s41598-020-61099-8, published online 09 March 2020

The Article contains an error in Table 1 where the Thickness of the polyimide layers (e & f) is incorrectly quoted.

The correct value should read 0.5 mm.

